# Application value of gastric contrast ultrasonography in gastric tumors

**DOI:** 10.1186/s13244-026-02242-3

**Published:** 2026-03-17

**Authors:** Guanmo Liu, Jie Li, Hua Liang, Zicheng Zheng, Chenggang Zhang, Yixuan He, Yihua Wang, Yang Gui, Weiming Kang, Xin Ye

**Affiliations:** 1 Department of General Surgery, Peking Union Medical College Hospital, Chinese Academy of Medical Sciences & Peking Union Medical College, Beijing, People’s Republic of China; 2 Department of Ultrasound, Peking Union Medical College Hospital, Chinese Academy of Medical Sciences & Peking Union Medical College, Beijing, People’s Republic of China

**Keywords:** Oral contrast ultrasonography, Double contrast-enhanced ultrasonography, Stomach neoplasms, Diagnosis, Treatment

## Abstract

**Abstract:**

In recent years, gastric oral contrast ultrasonography (OCUS) and double contrast-enhanced ultrasonography (DCEUS) have emerged as promising imaging techniques for evaluating gastric tumors, particularly gastric cancer (GC), by providing beneficial insights into tumor morphology, vascular characteristics and response to therapy. OCUS is capable of identifying the thickened gastric wall and lesions by utilizing oral contrast agents. DCEUS, based on OCUS, further employs intravenous contrast-enhanced ultrasound to examine regions of interest, thereby revealing both the anatomical features and perfusion characteristics of the lesions. These two methods are known for being non-irradiating, cost-effective, noninvasive and real-time. With advances in contrast agents and imaging technology, OCUS and DCEUS are expected to become increasingly reliable and contribute to improving patient prognoses. However, the clinical application of these techniques is still evolving, limited by a lack of technical standardization, operator dependence, and inadequate evaluation in certain tumor subtypes and patient populations. This review summarizes the current state in clinical practice of OCUS and DCEUS in the screening, diagnosis, staging and treatment assessment of gastric tumors. It also proposes possible orientations for clinicians to address the identified limitations and improve the clinical value of OCUS and DCEUS, including the integration of artificial intelligence and molecular imaging, alongside establishing international standardization protocols, enhancing physician training systems, and expanding accessibility to primary care settings. Through these efforts, OCUS and DCEUS may become indispensable components of future GC management strategies.

**Critical relevance statement:**

By summarizing gastric contrast ultrasonography across screening, diagnosis, T staging, N staging and treatment assessment in gastric tumors, and comparing with CT, EUS and endoscopy, this review proposes protocol standardization, operator training and AI-enabled quantification to advance its clinical application.

**Key Points:**

- Gastric tumors urgently require a simple, inexpensive, noninvasive and easily accessible screening method.
- Gastric contrast ultrasonography demonstrates utility in detecting and staging gastric tumors.
- Gastric contrast ultrasonography offers accessible and effective tools for screening of gastric cancer.
- Accelerating clinical application of gastric contrast ultrasonography demands standardization and technological innovation.

**Graphical Abstract:**

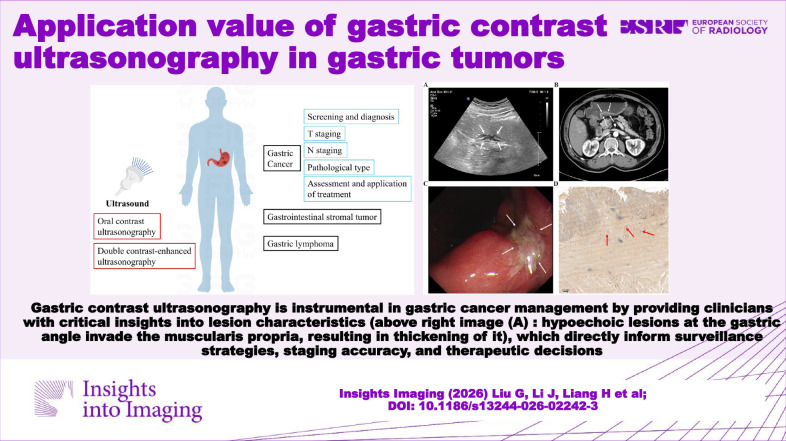

## Introduction

As a straightforward, repeatable, and convenient examination technique, ultrasonography is crucial for diagnosing illnesses. The technique possesses safety benefits, a non-irradiant nature, the capacity to deliver “real-time” information and prominent visualization and allows for detecting and describing malignancies and inflammatory processes [1]. Nonetheless, one of the challenges of ultrasonography is the evaluation of the digestive tract. The abundance of heterogeneous elements in the digestive lumen, like food debris and gas, obscures the lesions and peristaltic characteristics of the digestive tract, which are two major restrictions resulting in mistakes with erroneous positive or negative diagnoses. Transabdominal ultrasound assessment of the digestive tract can now be performed more effectively thanks to processes to refine the approach, like using contrast agents [2].

Gastroscopy, endoscopic ultrasonography (EUS), and CT are commonly used to diagnose gastric abnormalities [3–6]. Gastroscopy provides direct visualization of the stomach and allows for biopsy, making it a valuable tool for diagnosing gastric cancer (GC) with relatively high sensitivity and specificity. However, it is an invasive and costly procedure that requires general anesthesia to minimize discomfort [7]. Many patients, particularly the elderly, may be hesitant to undergo gastroscopy, which can lead to missed opportunities for timely treatment of gastric tumors [8]. Additionally, CT scans face challenges in detecting hypodense submucosal areas due to limited soft tissue resolution and the use of ionizing radiation, which restricts their application in clinical practice [9].

Recent advancements in ultrasound technology and contrast agents have made gastric contrast ultrasonography a valuable tool due to its radiation-free, noninvasive, and cost-effective nature. This method can enable dynamic monitoring of gastric lesions by filling the stomach with contrast agents [10, 11]. Oral contrast ultrasonography (OCUS) uses oral contrast agents to visualize the gastric wall and any lesions [12], while double contrast-enhanced ultrasonography (DCEUS) refers to the detection of gastrointestinal lesions based on OCUS, followed by contrast-enhanced ultrasonography (CEUS) performed on the region of interest, which can reveal the anatomical features of the gastric wall lesions and the perfusion features [13]. Gastroscopy, CT, and EUS remain the mainstream methods for examining gastric tumors. In contrast, the clinical use of gastric contrast ultrasonography is relatively limited, with few large-scale, high-quality studies available. However, as a potentially effective and feasible examination method for the future, we have comprehensively summarized the clinical value of gastric contrast ultrasonography in areas such as screening, diagnosis, staging and treatment assessment of gastric tumors. Additionally, we compare gastric contrast ultrasonography with other imaging methods used to monitor gastric tumors. This review aims to provide potential orientations for improving gastric contrast ultrasonography to promote its broader clinical application in gastric tumors.

## Techniques

### Gastric OCUS

OCUS for gastric imaging originated in 1978 when Warren et al first used a hydrophilic methylcellulose suspension to visualize abdominal organs [14]. This contrast agent improved the visualization of gastric and duodenal anatomy compared to water-filling alone [15], leading to subsequent further research on oral contrast agents for gastric ultrasound [16–21]. Due to the gastric wall being hypoechoic, and many lesions on the gastric wall, such as gastric cancer and ulcers, often present as hypoechoic as well, compared with hypoechoic or anechogenic contrast, mid-to-high echogenic contrast agents are now preferred for superior gastric wall visualization [22–24]. The technique involves filling the gastric cavity with the echogenic contrast agent to exhaust gas and food residue further, thereby improving lesion detection [8, 25].

OCUS enables detailed examination of the stomach wall’s five-layer structure and offers clearer visualization of the entire gastrointestinal wall layer and delineation of the relationship between lesions and surrounding organs [26, 27], which contributes to accurately assessing the extent and depth of lesion infiltration compared to gastroscopy and gastrointestinal barium examination. However, OCUS diagnostic accuracy for gastric filling can be compromised by obesity, flatulence and the examiner’s experience. Crucially, unlike endoscopy, OCUS does not allow for direct biopsy for histopathological examination or concurrent therapeutic intervention [12].

Several available contrast agents used in OCUS studies are commonly cellulose-based oral contrast agents, mainly consisting of bean and grain with sweet and pleasant tastes, which are generally acceptable to the patients [4, 5, 26, 28, 29]. According to most studies, participants must fast and abstain from fluids for at least 8 h and 4 h, respectively, to exclude the air in the gastric cavity as soon as possible [12, 25, 30–33]. As for the time interval available to perform the examination after the contrast agent swallowing, there is currently no clear mean time window according to the majority of studies [4, 5, 26, 30]. OCUS is usually performed immediately after drinking the oral contrast agent. The sonographer continuously images the patient from various positions and angles, systematically visualizing the cardia, fundus, body, antrum, duodenum, and abdominal esophagus in sequence [34–37].

### Gastric DCEUS

Perfusion imaging techniques are widely employed across oncologic specialties, providing quantitative imaging biomarkers that reflect tumor microvascular architecture and function [38–43]. DCEUS, integrating both intraluminal and intravenous contrast, enables a relatively comprehensive assessment of both the morphological features and vascular dynamics of normal and pathological gastric structures [44, 45]. Oral contrast agents fill the gastric lumen and empty intraluminal air, enhancing the visualization of mucosal lesions [46]. Intravenous contrast agents delineate the contours of the stomach wall and lesions, reveal their vascular characteristics, and highlight abnormal perfusion patterns [13].

Patients need to fast for 6–8 h before the exam [47–49]. The selection and preparation of oral contrast agents for DCEUS are similar to their counterparts in OCUS [22, 49–52]. The primary intravenous contrast agent is SonoVue [52], a microbubble agent containing sulfur hexafluoride [53, 54]. The contrast is eliminated via the lungs within 15 min post-injection and is therefore relatively safe for patients with poor renal function [55, 56]. Following blood circulation, the contrast reaches the capillary beds within the gastric lesions. Its microbubbles generate strong acoustic echoes across frequencies used in medical ultrasound [53, 57, 58].

Patients first ingest oral contrast agents and dynamic scanning of the stomach is conducted [47, 49, 59, 60]. Tumor dimensions, morphology, and echogenicity are documented, and subsequently, the appropriate dose of the contrast is injected intravenously [47, 48]. The whole process of contrast is recorded in the ultrasound instrument. The region of interest (ROI) is selected for quantitative analysis using acoustic density quantitative analysis software [52]. The acoustic density quantitative analysis software generates the time-intensity curve (TIC), records the arrival time (AT), peak intensity (PI), and basic intensity (BI), and calculates the enhanced intensity (EI) at the lesion and its peripheral gastrointestinal wall, respectively, with EI(dB) = PI(dB) − BI(dB) [50–52]. However, gastric motility, probe movements, and the patient’s breathing with the nearby diaphragm might affect the reliability of quantitative analysis according to the studies[22, 47, 61].

## Evaluation of gastric tumors

### Gastric cancer

#### Screening and diagnosis

GC, ranking as the sixth most common cancer and the fourth leading cause of global cancer-related deaths, imposes a significant disease burden and substantial healthcare costs [62]. China faces particularly severe challenges, grappling with one of the world’s highest GC incidence rates and suboptimal survival outcomes [63]. Early diagnosis and screening remain paramount for improving prognosis, driving the use of various imaging modalities, including endoscopy, EUS, barium studies, CT, MRI and ultrasound [64–66]. Notably, the gastric contrast ultrasonography procedure is generally well-tolerated. The oral contrast agent is designed to be palatable, and the examination itself is noninvasive and does not cause discomfort or pain, which contributes to high patient compliance, making it a viable option for large-scale screening in asymptomatic populations. This section reviews the clinical applications of OCUS and DCEUS in GC screening and diagnosis (Table 1). Notably, the study of a 48-patient DCEUS series reporting 88% accuracy corresponded to a wide 95% confidence interval, underscoring imprecision and the hypothesis-generating nature of such estimates pending larger, multicenter validation.

**Table 1:** Diagnostic performance of gastric contrast ultrasonography for gastric cancer in diagnosis

Study design	Patient number	Patient cohort	Method	Indicators		Gold standard	Ref.
Retrospective study	196	Patients with ≥ 60 years old and positive examination results	OCUS	Accuracy (%)	95	Gastroscopic biopsy	[8]
				Detection rate (%)	9		
				Sensitivity (%)	95		
				Specificity (%)	100		
Retrospective study	107	Patients with pathology-confirmed gastric lesions	DCEUS	Accuracy (%)	86	Postoperative pathology	[44]
				Sensitivity (%)	91		
				Specificity (%)	75		
Prospective study	82	Patients with GC or gastritis	CEUS	Accuracy (%)	92	Gastroscopic biopsy + Postoperative pathology	[82]
				Sensitivity (%)	97**		
				Specificity (%)	79**		
Retrospective study	1602	Patients with gastric lesions	OCUS	Detection rate (%)	57 (ESGC) 98 (AGC)	Gastroscopic biopsy	[4]
				Sensitivity (%)	95		
				Specificity (%)	79		
Prospective study	288	Patients with GC	OCUS	Detection rate (%)	67 (ESGC in T1a) 77 (ESGC in T1b) 100 (AGC)	Gastroscopic biopsy + Postoperative pathology	[5]
				Sensitivity (%)	91		
				Specificity (%)	96		
Prospective study	126	Patients with GC	OCUS	Detection rate (%)	78 (ESGC) 100 (AGC) 94 (Overall)	Postoperative pathology	[77]
Retrospective study	48	Patients with GC	DCEUS	Accuracy (%)	88	Gastroscopic biopsy	[81]

*OCUS* oral contrast ultrasonography, *DCEUS* double contrast-enhanced ultrasonography, *CEUS* contrast-enhanced ultrasonography, *ESGC* early-stage gastric cancer, *AGC* advanced gastric cancer

** *p* < 0.01

Although gastroscopy is the gold standard for the diagnosis of GC, there is no screening method recommended by the World Health Organization (WHO) as a large-scale population preliminary screening for GC because of many practical considerations at present [67, 68]. A large-scale screening program by Shen et al involving 14,969 participants (64.94% response rate) demonstrated 9.09% of positive predictive value (PPV), 99.96% of negative predictive value (NPV), 72.72% of sensitivity, 98.93% of specificity, and 100% diagnostic agreement with gastroscopy in OCUS examination [26]. Zheng et al [12] showed that there was no statistically significant difference in the sensitivity, specificity, PPV, NPV and accuracy of gastric lesions detected by OCUS and gastroscopy through a multicenter large sample (383,945 patients included).

From 2007 to 2016, the national large sample about GC data in Korea suggested that the sensitivity of gastroscopy only increased from 66.4% (2007–2008) to 69.3% (2015–2016) [69]. OCUS has a higher detection rate for lesions (including dysplasia and canceration) showing hypoechoic gastric wall thickening. However, OCUS demonstrated a lower detection rate for gastric polyps, although the vast majority of those detected are benign, non-cancerous lesions, predominantly fundic gland polyps and hyperplastic polyps [31, 70–72]. Gastroscopy can sensitively find GC with gastric mucosal changes even at the millimeter level, but it may miss the diagnosis of advanced GC with obvious thickening of the gastric wall [4, 73]. OCUS is more acceptable with non-invasiveness and painlessness compared to the gastroscopy for screening and economical reasons. Therefore, it is also of positive significance to carry out OCUS as screening for improving the prognosis of GC, which is still under further exploration [4, 5, 26]. In practice, OCUS is best positioned as a front-end and pre-endoscopic screening and triage tool in high-incidence settings with limited endoscopic resources, in patients who are unwilling or temporarily unfit for gastroscopy, and in non-obese individuals with adequate acoustic windows. In such contexts, OCUS can cost-effectively flag suspicious focal wall thickening or deformity for targeted endoscopy, thereby reducing unnecessary invasive exams while preserving sensitivity for clinically relevant diseases [32, 61, 74] and complementing national endoscopic screening where resources or acceptance are restricted [26, 75]. By contrast, DCEUS adds its primary value as a problem-solving and screening adjunct. Using an intravenously administered, lung-cleared and non-nephrotoxic microbubble contrast agent [76], DCEUS can further characterize ambiguous gastric wall thickening identified on OCUS. If OCUS is negative but clinical risk remains, endoscopy should proceed in accordance with local policy. Notably, DCEUS is still an adjunct in many centers, underscoring the need to tailor adoption to local expertise and resources [75].

Clinical studies validate OCUS’s diagnostic capability. Liu et al reported high sensitivity and specificity for GC detection in elderly patients (*n* = 196), with excellent agreement versus gastroscopy (kappa = 0.972) [8]. Wu et al demonstrated OCUS superiority in detection rate over enhanced CT for both early-stage GC (ESGC) and advanced GC (AGC) in the study enrolling 126 patients, but there was no statistically significant difference between the two examinations for AGC in detection rate [77]. However, diagnostic accuracy is influenced by body habitus. Significantly higher detection rates occurred in patients with favorable anatomy (clear visualization of cardia or pylorus) than in unfavorable cases (100% vs. 83%, *p* < 0.05) [5]. OCUS is thus particularly recommended for non-obese individuals with suitable anatomy [70, 78, 79].

Furthermore, studies indicated that gastric wall thickness on OCUS correlates with malignancy risk. Guan et al found significantly higher GC incidence in patients with wall thickness > 9 mm (*p* < 0.01) [25]. Liu et al [4] developed the Stomach Ultrasound Reporting and Data System (Su-RADS): (1) Category 2 (1.5–2 mm of mucosal thickness): 1.7% malignancy risk; (2) Category 3 (2–2.5 mm of mucosal thickness): 12.2% malignancy risk; (3) Category 4 (2.5–5 mm of mucosal thickness): 34.2% malignancy risk; (4) Category 5 (> 5 mm of mucosal thickness): 78.1% malignancy risk. Sensitivity and specificity of using Su-RADS (cutoff: category 2) reached 95.1% and 78.6%, respectively. Whereas, Su-RADS is only limited to this study presently, and whether it can be widely used still needs to be further studied with a larger sample. Integrating risk stratification systems like Su-RADS with artificial intelligence (AI)-driven automation possibly holds significant promise for optimizing early detection and clinical decision-making. Sui et al’s U-net model, which was a combination of AI, achieved 95% accuracy in gastric layer segmentation and lesion classification (AUC = 0.92) [80]. However, it should be mentioned that Su-RADS in this study inevitably overlooked some ESGCs that did not manifest as stomach wall thickening, and certain demographic biases existed [4].

DCEUS improves diagnostic precision by enhancing microvascular imaging through intravenous contrast, aiding lesion characterization. Zhou et al reported better diagnostic accuracy of AGC for DCEUS than MRI, with statistical differences [81]. Malignant lesions typically exhibit delayed, heterogeneous enhancement with irregular margins and perigastric fat infiltration, reflecting abnormal vascular architecture [82–85]. Quantitative analyses revealed distinct parameters: (1) Shorter AT with GC: 8.68 ± 2.06 s (GC) vs. 10.43 ± 2.75 s (normal tissue; *p* = 0.017) [13, 44]; (2) Higher PI and EI with GC: PI = 34.64 ± 6.63 dB vs. 29.58 ± 8.22 dB (*p* = 0.023); EI = 29.72 ± 6.69 dB vs. 22.66 ± 7.01 dB (*p* = 0.001) [13]. Delayed washout patterns further supported malignancy diagnosis [60]. Notably, EI strongly correlated with pathological microvessel density (r = 0.921, *p* < 0.001), possibly serving as a potential biomarker for angiogenesis [86].

#### Staging

##### T staging

Pathological staging critically influences long-term survival in GC. The 5-year survival rate is 90–95% for stage T1 GC but drops sharply to 7–27% for AGC [87]. Accordingly, the NCCN guidelines (v2.2022) recommend perioperative chemotherapy for stage T2 and above or lymph node-positive cases [87]. This underscores the clinical imperative for precise preoperative staging through imaging modalities to guide personalized treatment strategies [88–91].

**Fig. 1:**
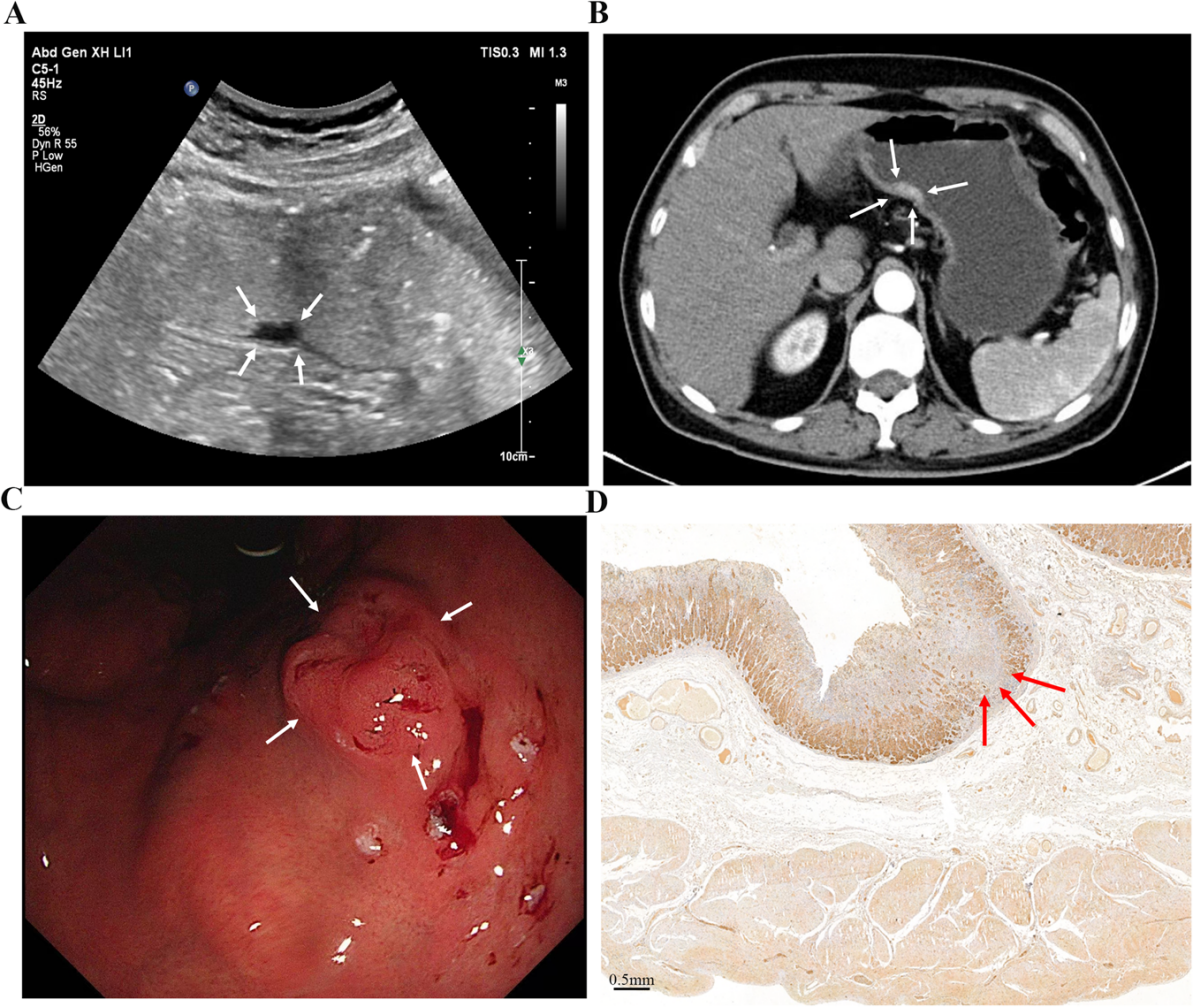
**A** An approx. 65-year-old man underwent gastric oral contrast ultrasonography, revealing a low echo in the mucosal layer characterized by an irregular shape on the lesser curvature of the gastric body (cT1). **B** The tumor showed local thickening and enhancement, indicating invasion of the muscularis propria layer of the posterior wall (cT2) in the lower part of the gastric body on enhanced computed tomography. **C** Electronic gastroscopy found a protrusion lesion on the lesser curvature of the gastric body, with intermediate ulcer formation. **D** The pathological result showed that the tumor invaded the submucosa (pT1) (scale bar: 0.5 mm)

**Fig. 2:**
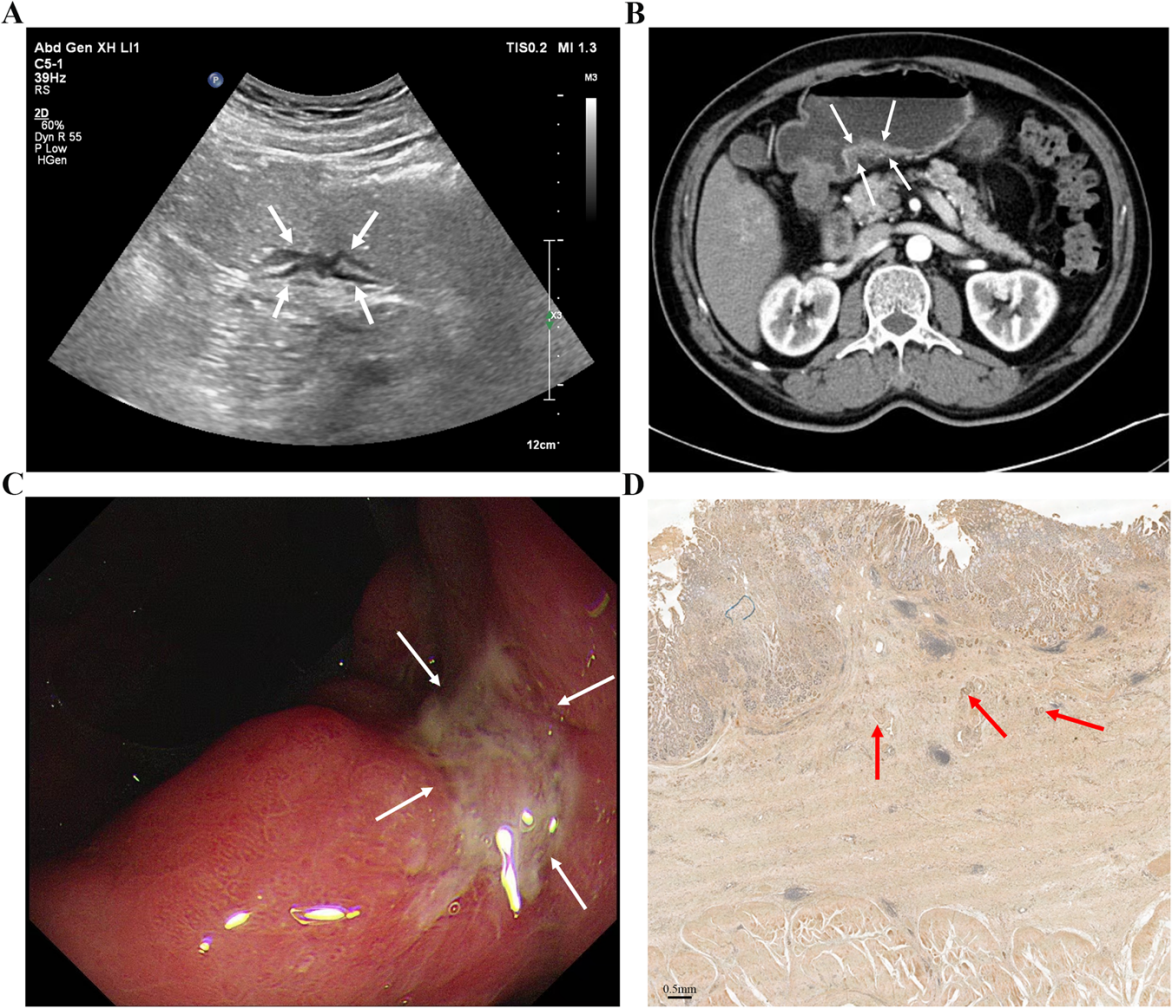
**A** An approx. 55-year-old man underwent gastric oral contrast ultrasonography, revealing hypoechoic lesions at the gastric angle that invaded the muscularis propria layer (cT2). The muscularis propria layer was thickened. The gastric wall was destroyed, and the serosa remained intact. **B** The tumor showed local thickening and enhancement, indicating invasion to the subserosal layer (cT3) of the posterior wall of the gastric antrum on enhanced computed tomography. **C** Electronic gastroscopy found a mucosal lesion in the gastric angle with a formed central ulcer and a sense of mucosal convergence. **D** The pathological result showed that the tumor invaded the muscularis propria layer (pT2) (scale bar: 0.5 mm)

**Fig. 3:**
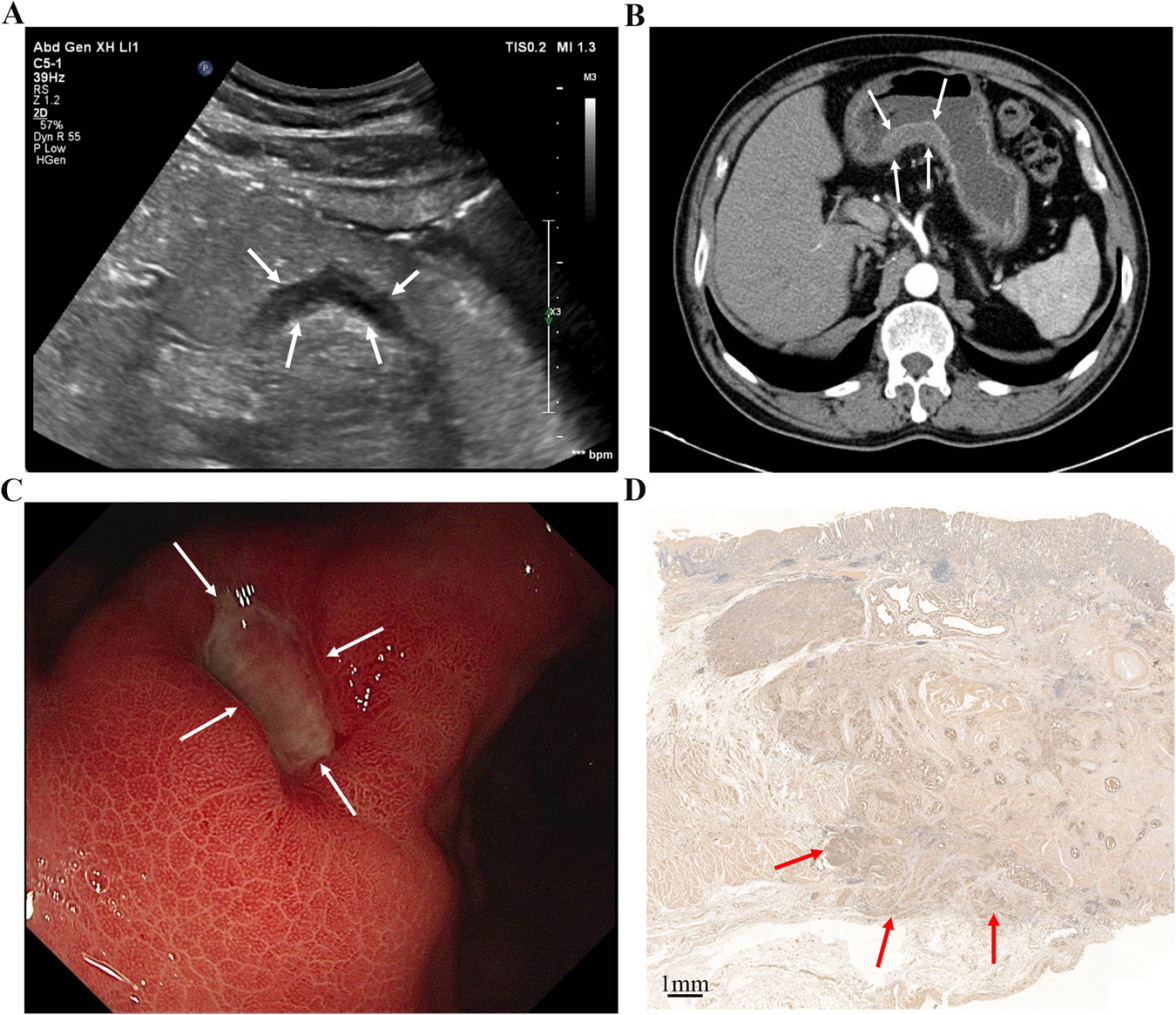
**A** An approx 70-year-old man underwent gastric oral contrast ultrasonography, revealing that low echo invaded the subserosal layer (cT3) at the gastric angle. The hierarchical structure of the gastric wall was unclear. The mucosa presented as rough, and the strong echogenic zone of the serosa was not smooth. **B** The tumor showed local thickening and enhancement, indicating invasion to the subserosal layer (cT3) of the lower part of the gastric body on enhanced computed tomography. **C** Electronic gastroscopy found a mucosal lesion in the gastric angle, with central ulcer formation. **D** The pathological result showed that the tumor invaded the muscular layer of the gastric wall to the surrounding adipose tissue, not involving the serosa (pT3) (scale bar: 1 mm)

**Fig. 4:**
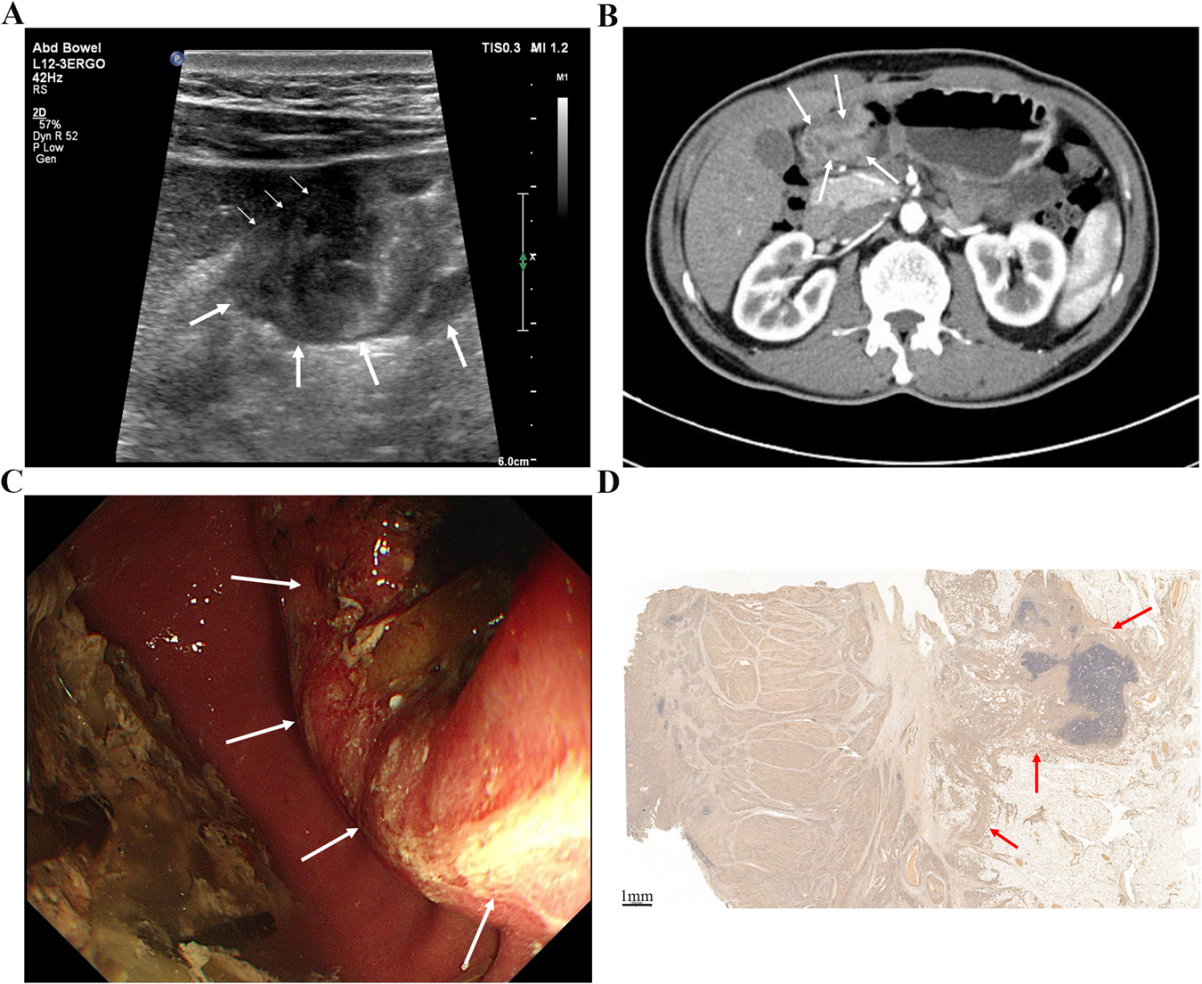
**A** An approx 55-year-old man underwent gastric oral contrast ultrasonography, revealing full-thickness thickening of the gastric wall (cT4) at the gastric antrum (white thick arrows), with irregular shape, low internal echo, destruction of the gastric wall, irregular serosa echo line, gastric peristalsis stiffness, and unclear boundary with liver tissue (white thin arrows). **B** The tumor showed local thickening and moderate enhancement, indicating invasion of the whole layer (cT4) of the gastric antrum on enhanced computed tomography. **C** Electronic gastroscopy found a protrusion ulcer lesion on the gastric antrum, with a white moss-covered center. **D** The pathological result showed that the tumor invaded the whole layer of the gastric wall and the serosa (pT4) (scale bar: 1 mm)

GC typically originates in the mucosa, progressing through the submucosa, muscularis propria, and serosa. This often appears as gastric wall thickening or masses, forming the basis for T staging via gastric contrast ultrasonography [92]: (1) 0: no detectable tumor; (2) T1: tumor limited to mucosa/submucosa; (3) T2: muscularis propria invasion with intact serosal hyperechoic band; (4) T3: serosal penetration with irregular hypoechoic contours and discontinuous serosal layer; (5) T4: perigastric fat/organ invasion, disrupted serosal line [93]. In a small single-center cohort (*n* = 42), He et al reported 86% overall T-stage accuracy (36/42 cases) using OCUS, highest for T4 (98%), followed by T2 (88%) and T3 (86%) [29]. However, T1 was excluded from this study. Given the limited sample and spectrum restrictions, these estimates should be regarded as preliminary. Another study showed variation compared to this study, with lower accuracy for T1/T2 (95%) versus 100% for T3/T4 [27], likely due to nonspecific lesions and ambiguous mucosal-muscular boundaries. Combined imaging may improve precision. Gai et al, enrolling 109 patients, indicated sensitivity, specificity and accuracy of preoperative T staging evaluation reached 88–100% by combined detection of the three methods (OCUS, CT and MRI) [94]. OCUS tends to evaluate submucosal and propria invasion well, while CT excels at assessing serosal and perigastric involvement [29, 77, 95, 96]. Yu et al found that combining OCUS and CT increased T staging accuracy to 85% versus 75–77.5% for a single examination [97]. Furthermore, OCUS based on AI could better help to judge the lesion process, depth of invasion, and lymph node metastasis [98]. Figures 1–4 demonstrate lesion images from OCUS, enhanced CT, and gastroscopy examination in GC patients with different T staging, along with corresponding postoperative pathological images. OCUS findings (Fig. 1A–4A) corroborated pathological confirmation (Figs. 1–4D) and aligned with CT (Figs. 1–4B) or gastroscopy (Figs. 1–4C) results.

DCEUS improves tumor localization, margin characterization, and enhancement analysis and T staging relies on strong arterial hyper-enhancement and rapid venous washout patterns, creating a ‘negative display’—manifested as narrowing, disappearance, or destruction of the gastric wall—compared to normal tissue [24, 45, 50, 53, 99, 100]. DCEUS focuses on angiogenesis (a malignancy hallmark) and enhances staging utility beyond size or morphology [101, 102]. While Li et al reported over 80% DCEUS accuracy for T2-T4 stages [53], accuracy varied by location, with approximately 92% for the gastric antrum and pylorus, about 85% for the body and fundus, and around 69% for cardia, likely due to differences in oral contrast filling [103]. Compared to OCUS, DCEUS consistently outperformed it for T2-T4 stages [53, 104, 105], though T1 accuracy was comparable between the two examinations [106]. A meta-analysis by Zhong et al also demonstrated that DCEUS and OCUS performed equally in distinguishing T1 from lesions with T2 and above [9]. Considering the absence of contrast-related risks and its lower cost, OCUS could be considered a preferable modality for ESGC in specific clinical scenarios in comparison to DCEUS [107, 108]. To establish its role as an auxiliary tool for T staging, however, more evidence from large-scale studies is needed to validate in diagnostic precision.

**Fig. 5:**
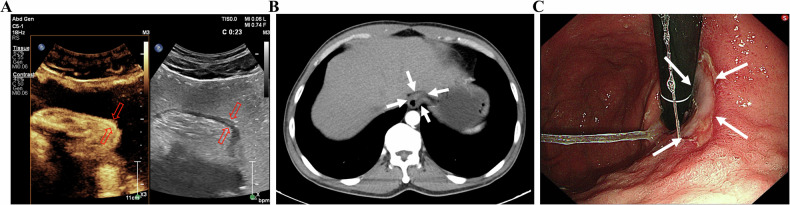
**A** An approx 55-year-old man underwent gastric double contrast-enhanced ultrasonography and oral contrast ultrasonography, revealing a subserous layer significantly enhanced in the arterial phase (left) and a low echo invaded the subserosal layer (cT3) at the cardia (right), respectively. The hierarchical structure of the gastric wall was unclear. The mucosa presented as rough, and the strong echogenic zone of the serosa was not smooth. **B** The tumor showed local thickening and enhancement, indicating invasion to the subserosal layer (cT3) of the cardia and the mall curvature side of the gastric body on enhanced computed tomography. **C** Electronic gastroscopy found a large and deep ulcer on the lesser curvature of the cardia

EUS was also significantly superior to DCEUS for T1, and DCEUS was more accurate than EUS for T3 and T4 [22, 99]. EUS is difficult to differentiate fibrosis and inflammation from tumors, and thus, the accuracy of EUS in late-stage tumors is reduced. Contrariwise, DCEUS is better for determining the staging of AGC due to the rich blood supply. The lack of vascularity in ESGC leads to its low accuracy [99]. However, when compared with enhanced CT, DCEUS evaluated T1 better, whereas CT was superior to DCEUS in the evaluation of T3 and T4 GC [6, 48, 50, 97, 109], because CT might, to some extent, more easily identify the perigastric fat plane around the tumor (T3), irregular or nodular borders, or direct invasion of the adjacent organs(T4) [97, 109]. DCEUS and CT have complementary strengths or weaknesses for T staging, depending on GC stage, location, and patient factors. Clinicians should select the optimal method accordingly. Table 2 presents the diagnostic performances of OCUS and DCEUS for T staging of GC, comparing them with enhanced CT, MRI, and EUS in detail, as well as comparing OCUS versus DCEUS. Figure 5 shows the lesion images of one GC patient from the different examinations evaluating T staging (DCEUS, enhanced CT and gastroscopy, respectively).

**Table 2:** Diagnostic performance of gastric contrast ultrasonography and comparisons with other imaging methods for gastric cancer in T staging

	Study design/patient number	Method	Indicators	T1		T2		T3		T4		T Total		Ref.
Comparison with CT	Retrospective/54	DCEUS		Ultrasonography	CT	Ultrasonography	CT	Ultrasonography	CT	Ultrasonography	CT	Ultrasonography	CT	
			Accuracy (%)	98	92	85	89	76	83	85	94	—	—	[48]
			Sensitivity (%)	88	75	43	57	89	89	42	83	—	—	
			Specificity (%)	100	96	91	94	63	78	98	98	—	—	
	Retrospective/60	OCUS	Accuracy (%)	—	—	88	93	86	83	98	90	86	83 ^ns^	[29]
			Sensitivity (%)	—	—	25	50	77	62	100	100	—	—	
			Specificity (%)	—	—	95	97	90	93	94	76	—	—	
	Prospective/109	OCUS	Accuracy (%)	94	87	90	83	90	82	95	92	94	84*	[94]
			Sensitivity (%)	80	60	84	68	86	72	82	76	—	—	
			Specificity (%)	98	95	92	88	92	86	98	95	—	—	
	Prospective/108	OCUS	Accuracy (%)	84	53*	82	73^ns^	69.4	88*	66	72 ^ns^	72	76 ^ns^	[77]
	Prospective/229	DCEUS	Accuracy (%)	93	70***	73	52*	86	46***	88	70*	87	61***	[50]
Comparison with EUS	Retrospective/158	DCEUS		Ultrasonography	EUS	Ultrasonography	EUS	Ultrasonography	EUS	Ultrasonography	EUS	Ultrasonography	EUS	
			Accuracy (%)	63	84*	84	82^ns^	88	72*	91	65*	82	77 ^ns^	[22]
	Retrospective/162	DCEUS	Accuracy (%)	62	79^ns^	76	82^ns^	86	68*	93	67 ^ns^	77	75 ^ns^	[99]
Comparison with MRI	Prospective/109	OCUS		Ultrasonography	MRI	Ultrasonography	MRI	Ultrasonography	MRI	Ultrasonography	MRI	Ultrasonography	MRI	
			Accuracy (%)	94	87	90	83	90	87	95	93	94	89*	[94]
			Sensitivity (%)	80	68	84	71	86	78	82	76	—	—	
			Specificity (%)	98	93	92	88	92	92	98	96	—	—	
DCEUS VS. OCUS	Retrospective/81	—		DCEUS	OCUS	DCEUS	OCUS	DCEUS	OCUS	DCEUS	OCUS	DCEUS	OCUS	
			Accuracy (%)	99	96^ns^	96	95*	90	85**	90	85***	—	—	[106]
			Sensitivity (%)	—	—	67	56	62	43	76	65	—	—	
			Specificity (%)	—	—	96	96	94	87	96	78	—	—	
	Retrospective/62	—	Accuracy (%)	67	67	80	60	90	77	100	71	88	73*	[105]
	Prospective/350	—	Accuracy (%)			83	70	90	80	87	68	87	75***	[53]

*OCUS* oral contrast ultrasonography, *DCEUS* double contrast-enhanced ultrasonography, *CT* computed tomography, *EUS* endoscopic ultrasonography, *MRI* magnetic resonance imaging

* *p* < 0.05, ** *p* < 0.01, *** *p* < 0.001, ns: no significance

#### N staging

**Fig. 6:**
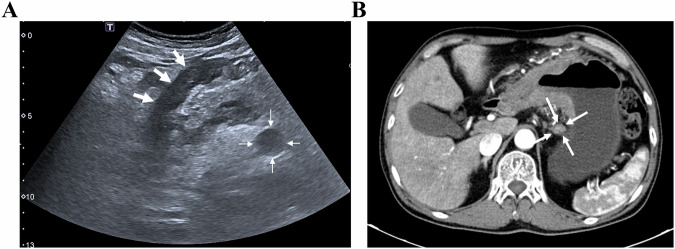
**A** An approx 60-year-old man underwent gastric oral contrast ultrasonography, revealing full-thickness thickening of the gastric wall of the gastric antrum (white thick arrow), multiple lymph node enlargement around (white thin arrow). **B** Multiple lymph node enlargement at the lesser curvature of the stomach (N3) on enhanced computed tomography

Lymph node metastasis (LNM), the primary metastatic pathway in GC, critically impacts prognosis and determines neoadjuvant therapy (NAT) use [110]. Blind lymph node dissection risks over-treating non-metastatic nodes, impairing immunity and increasing the surgical rate of complications [111, 112]. Therefore, accurate LNM assessment is essential for precision treatment. The assessment accuracy of LNM by conventional methods (transabdominal US, EUS, CT, PET-CT) remains unsatisfactory [20, 113–120]. Currently, the clinical application value of OCUS and DCEUS for LNM of GC is also being explored. Wang et al reported 95% accuracy of OCUS for all N stages (N0-N3), comparable to CT [27]. A combination of OCUS and CT yielded a superior diagnostic accuracy of 92.7%, compared to 85.3% for OCUS and 80.7% for CT when used individually in a study (*n* = 150) [121]. The combination of the two may be helpful and complement each other to enhance the accuracy of LNM diagnosis before operation, though the number of patients included in the study is small, and the current evidence is also limited. Figure 6A presents the OCUS image for the assessment of GC patients with LNM, along with the corresponding CT image (Fig. 6B).

As mentioned previously, angiogenesis also greatly contributes to the tumor’s LNM, including GC [122–127]. DCEUS can effectively evaluate the angiogenesis and malignant biological behavior of GC, and show hyper-enhancement compared with the adjacent normal gastric wall during the early arterial phase when LNM [86]. Although the specificity of DCEUS (60.7%) was lower than that of OCUS (98.4%), the sensitivity of DCEUS (86.7%) was significantly higher than that of OCUS (33.3%) in assessing LNM [23]. Furthermore, Li et al evaluated the predictive power of EI by DCEUS for LNM of GC [128]. In contrast with non-hyper-enhanced cases, in cases with hyper-enhanced GC, the tumor invasion was deeper, with worse differentiation and a higher rate of LNM. Compared with EUS, DCEUS had an elevated accuracy of 78.4% in the diagnosis of LNM (57.4% for EUS), which showed DCEUS might offer an advantage in lymph node detection, although this was a single-center retrospective cohort (*n* = 162), which might limit generalizability [99].

#### Pathological type

The Borrmann classification, established in 1926 and based on gross AGC morphology [129], defines four subtypes and remains a key prognostic tool [45, 130–132]. The primary investigative method for GC patient diagnosis is endoscopy, but for the Borrmann classification, it may be inappropriate [133]. Accurate preoperative classification is crucial for optimal treatment [47]. DCEUS achieved notable accuracy (91.49%) for Borrmann typing with excellent reproducibility (kappa = 0.844) [45], outperforming OCUS [132]. This is likely due to clear arterial-phase enhancement and venous-phase washout delineating tumor margins and depth. While OCUS detects diffuse wall thickening and masses well [45, 134–138], it struggles with micro-invasion and layer changes.

In contrast with enhanced CT, Yan et al indicated that the accuracy of DCEUS in assessing Borrmann classifications I, II, and III was higher than that of enhanced CT, but was lower in Borrmann classification IV [100], largely consistent with Xu et al [50], except Borrmann classification IV. However, in the study by Yan et al, both DCEUS and enhanced CT exhibited excellent detection rates for Borrmann classifications I and IV. This may be attributed to the characteristic morphological features of these two types. Borrmann classification I is characterized by nodular polypoid tumors, while Borrmann classification IV presents diffuse thickening of the gastric wall [100]. He et al conducted a similar study, and in this study, the diagnostic efficacy of DCEUS and enhanced CT in Borrmann classifications I, II, and III was comparable [47]. In conclusion, DCEUS is probably a valuable alternative for Borrmann classification in patients avoiding radiation, renal function burden from contrast agents, or cost constraints. Table 3 details the diagnostic performance of DCEUS for the Borrmann classification in GC and compares it with enhanced CT and OCUS.

**Table 3:** Diagnostic performance of gastric contrast ultrasonography and comparisons with CT for gastric cancer in Borrmann classification

Study design	Patient number	Indicators	Borrmann I		Borrmann II		Borrmann III		Borrmann IV		Ref.
Retrospective study	54		DCEUS	CT	DCEUS	CT	DCEUS	CT	DCEUS	CT	
		Accuracy (%)	96	94	87	80	72	74	89	96	[47]
		Sensitivity (%)	100	50	17	33	90	73	25	88	
		Specificity (%)	96	96	96	85	50	75	100	98	
Prospective study	239	Accuracy (%)	87	84^ns^	87	80***	87	80***	91	97^ns^	[100]
Prospective study	229	Accuracy (%)	100	71	82	48	83	61	88	56	[50]
Retrospective study	162		DCEUS	OCUS	DCEUS	OCUS	DCEUS	OCUS	DCEUS	OCUS	
		Accuracy (%)	91	89	92	77	91	76	92	82	[132]
Retrospective study	329		DCEUS		DCEUS		DCEUS		DCEUS		[45]
		Accuracy (%)	87		90		92		93		
		Sensitivity (%)	87		90		92		93		
		Specificity (%)	99		97		93		97		

*OCUS* oral contrast ultrasonography, *DCEUS* double contrast-enhanced ultrasonography, *CT* computed tomography

*** *p* < 0.001, ns no significance

Lauren’s classification divides GC into intestinal, diffuse, mixed, and unclassified types [139, 140]. Intestinal type (WHO classification: well or moderately differentiated) generally has a better prognosis than diffuse type (WHO classification: poorly differentiated, undifferentiated or signet ring) [141]. A study on DCEUS examined 34 patients with the intestinal type of lesions. Out of these, 30 lesions exhibited uniform enhancement following the intravenous injection of a contrast agent. In contrast, among 24 patients with the diffuse type, 22 lesions demonstrated uneven enhancement. The difference in the occurrence of inhomogeneous enhancement between the two Lauren subtypes was statistically significant [24]. DCEUS enhancement heterogeneity might be suggestive of diffuse type in appropriate clinical contexts, but given the small sample size and single-center design of this study, this signal should be regarded as preliminary and hypothesis-generating, not a stand-alone diagnostic criterion. There are a few relevant studies presently, pending further confirmation in larger, prospective, multicenter cohorts.

In addition, for differentiated and undifferentiated GC, Wei et al found that the most common DCEUS pattern in differentiated GC was homogeneous enhancement, while the most common DCEUS pattern in undifferentiated GC was inhomogeneous enhancement [11]. There was a significant difference in the proportion of heterogeneous enhancement between the two histological subtypes. Tumor vascularization can be assessed using contrast agents, and studies have shown that contrast enhancement correlates with histological vascular density [142, 143]. In comparison to the differentiated type, the undifferentiated type exhibits fewer blood vessels and more diffuse infiltration [96, 144], which may explain the findings of Wei et al.

#### Assessment and application of treatment

NAT, including chemotherapy, radiotherapy, immunotherapy and targeted therapy, is currently the standard treatment for local AGC, improving resectability and survival [145–147]. However, response varies, necessitating accurate prediction and assessment to guide further treatment. Current NAT response assessment via CT using RECIST criteria is limited for gastric tumors due to organ motility/filling variations, leading to poor prognostic correlation [148]. Therefore, preoperative NAT requires an accurate and noninvasive technique to accurately assess treatment response. A small study of patients receiving NAT reported PPV and NPV of 70.8% and 47.4% for DCEUS in distinguishing responders from non-responders, with apparent higher accuracy than CT with the RECIST standard in that cohort [10]. In view of the small, single-center nature of the available cohorts (*n* = 43), these results might be regarded as preliminary and validated prospectively before broader application. Another study compared the DCEUS parameters of responders and non-responders before and after NAT. PE (peak enhancement) and TP (time to peak) after NAT, and the ratio of ΔPE/ΔTP (change ratio of PE and TP) was significantly different between the two groups. The study showed that ΔPE was superior to other parameters in evaluating the responsiveness of GC patients to NAT, and its sensitivity and specificity for evaluating the therapeutic effects of NAT were 82.7% and 64.9% in GC, respectively [149]. Moreover, a study used the cutoff value of CEUS to predict the expression of programmed death ligand-1 (PD-L1) and found that there was an elevated correlation between CEUS results and PD-L1 expression in GC. It was indicated that CEUS parameters could be used as predictors of treatment response of immune checkpoint inhibitors in the clinical practice of NAT in GC [150].

### Gastrointestinal stromal tumor

**Fig. 7:**
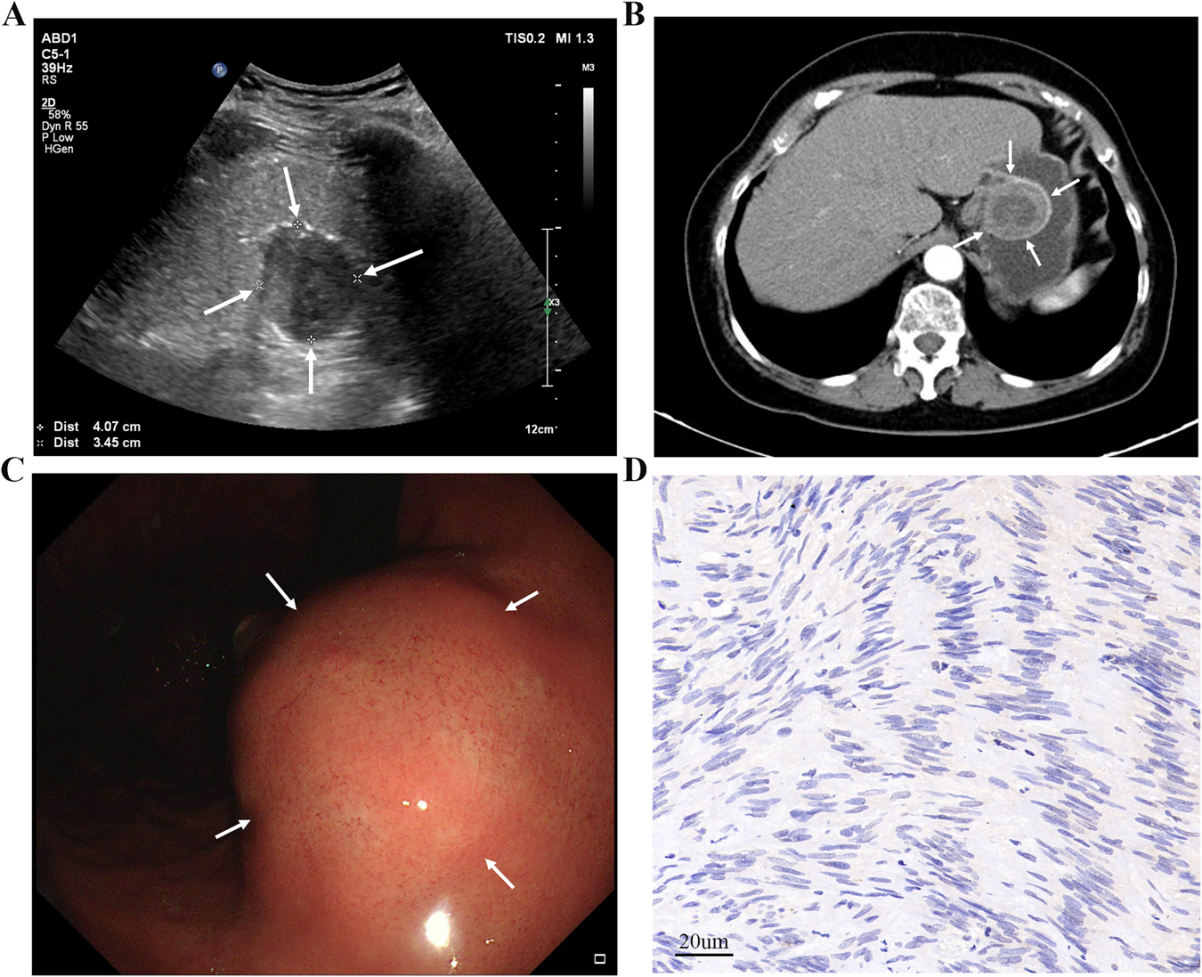
**A** An approx 75-year-old woman underwent gastric oral contrast ultrasonography, revealing a heterogeneous low echo under the gastric fundus mucosa, which was round and about 4.1 × 3.5 cm. The shape was regular with a smooth surface, and the boundary was clear. The hierarchical structure of the gastric wall was clear and not obviously thickened. **B** Submucosal round cystic solid mass at the fundus of the stomach, with clear boundaries and convex shape toward the gastric cavity, about 4.3 × 4.0 cm, presented enhanced scan with mild uneven enhancement on enhanced computed tomography. **C** Electronic gastroscopy found a 4.0 × 5.0 cm submucosal bulge in the fundus of the stomach. The surface was smooth, and the color was the same as the surroundings. **D** The pathological result showed that the gastrointestinal stromal tumor was 4.6 × 4.5 × 3.5 cm (scale bar: 20 μm)

Gastrointestinal stromal tumors (GISTs), the most common mesenchymal neoplasms of the GI tract, exhibit an annual incidence of 4.3–22.0 cases/million globally [151, 152], with rising trends reported in recent decades [153, 154]. High-risk GISTs face elevated recurrence or metastasis risks despite complete surgical resection remaining the curative standard [155–159]. Noninvasive preoperative imaging and risk stratification are thus critical for treatment planning and surveillance. OCUS can clearly show that it originates from the submucosa or muscularis propria layer. GISTs are usually presented as round, oval, or lobulated in shape, and they may exhibit either uniform or uneven low echogenicity. The boundary is clear and large stromal tumors are often accompanied by liquefaction necrosis and calcified plaques in OCUS [44, 51]. Figure 7A displays the image of gastric abnormality identified as GIST by OCUS, which is consistent with its pathological results (Fig. 7D). The corresponding images of CT (Fig. 7B) and gastroscopy (Fig. 7C) are also provided.

**Fig. 8:**
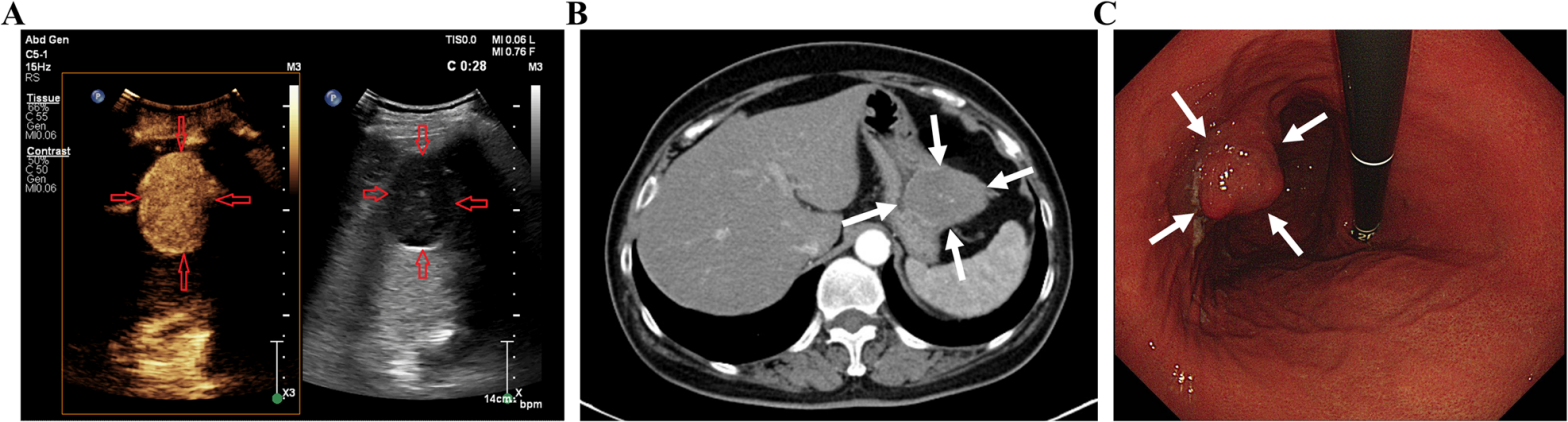
**A** An approx 65-year-old woman underwent gastric double contrast-enhanced ultrasonography and oral contrast ultrasonography, revealing a heterogeneous hyper-enhancement (left) and low echo under the gastric fundus mucosa(right), respectively, which was round and about 5.5 × 3.5 cm. The shape was regular with a smooth surface, and the boundary was clear. The hierarchical structure of the gastric wall was clear and not thickened. **B** Ellipsoid-shaped mass was seen on the greater curvature of the gastric body, with a size of about 56 × 38 mm, a clear boundary and partial lobes at the edge. After enhancement, it showed uniform and progressive enhancement on enhanced computed tomography. **C** Electronic gastroscopy found a diameter of about 3 cm submucosal bulge in the greater curvature side at the junction of the gastric fundus and the gastric body

Shi et al [13] and Stock et al [143] manifested that when given an intravenous contrast agent, the profile of GIST following the contrast agent was represented by early, intense, homogeneous enhancement followed by a slow washout of the contrast agent. In the study of Li et al, however, 64.3% cases demonstrated homogeneous hypo-enhancement or hyper-enhancement, and other cases were heterogeneous hypo-enhancement or hyper-enhancement centrally within the lesion [44]. The current research on the comparison between DCEUS and other examinations in GIST is limited. In a study involving 139 lesions conducted by He et al, DCEUS successfully identified 120 lesions, while EUS identified 113 lesions [51]. There was no significant difference in accuracy between the two methods, which might suggest that DCEUS can not only display the location, size, shape, boundary, and internal echo of GIST like EUS, but also provide information about the blood perfusion patterns of GIST. Figure 8 depicts DCEUS characteristics of the GIST (Fig. 8A), with corresponding features on enhanced CT (Fig. 8B) and gastroscopy (Fig. 8C). At present, studies on OCUS in GISTs are still rare.

GIST typically has a more extensive vascular network than GC [160], and the related parameters of GC and GIST are also different in DCEUS. PI is linked to the maximum dose of the contrast agent that reaches the lesion and is proportional to the average blood flow in the ROI, as we know. Therefore, one would expect the PI for GIST to be higher than that for GC. However, recent studies have shown that the PI for GC was higher than that for GIST during DCEUS. This discrepancy may be due to GIST being more susceptible to hemorrhage and necrosis, which can lead to reduced internal blood flow and consequently lower concentrations of the contrast agent within the tumor [52]. Furthermore, in DCEUS, the AT of the contrast agent in the GC group was found to be quicker than in the GIST group [44, 52]. Unlike GCs, most GISTs are discovered in the muscular layer of the stomach. The blood flow from both the mucosal and serosal layers contributes to the vascular supply of the muscle layer where GIST is located, creating a blood perfusion pattern that flows from the outer edges toward the center. This characteristic results in the slower progression of GIST compared to GC in AT.

Early identification of high-risk GIST patients remains a major challenge in current clinical practice. The expression of vascular endothelial growth factor (VEGF) is higher in high-risk GIST than in low-risk GIST [161]. VEGF plays a critical role in the formation of new blood vessels, suggesting that individuals in high-risk groups tend to have a greater number of these new vessels. The size, AT and PI of GIST assessed through DCEUS hold potential as parameters in a preoperative, noninvasive risk classification prediction model for GIST [49]. In comparison to low-risk GIST, high-risk GIST exhibited faster AT, larger sizes, and higher PI. These findings aligned with results from another study [52]. A study also revealed that high-risk GIST on CEUS showed arterial hyperperfusion that progressed slowly from the edge to the center [143]. Additionally, Li et al demonstrated significant cutoff values for DCEUS parameters that predicted malignant potential: a size of 2.4 cm, an AT of 9.04 s, and a PI of 15.2 dB [49].

### Gastric lymphoma

Primary gastric lymphomas account for approximately 5% of all gastric malignancies, representing a rare subset of gastric tumors [162]. Histologically, they are broadly classified into two categories: low-grade mucosa-associated lymphoid tissue (MALT) lymphoma and high-grade diffuse large B-cell lymphoma (DLBCL) [162]. Current clinical guidelines from the Chinese Society of Clinical Oncology and European Society for Medical Oncology recommend CT and PET-CT as primary modalities for therapeutic monitoring and response assessment [163, 164]. However, their accessibility is limited by substantial radiation exposure and high operational costs. OCUS has also been used in some studies to evaluate gastric lymphomas, plausibly emerging as a cost-effective and non-radiation alternative. OCUS demonstrates unique capabilities in gastric lymphoma evaluation. Distinct imaging profiles differentiate MALT lymphoma and DLBCL. Superficial-type presentations dominated MALT cases (66.67%), while diffuse infiltrative patterns characterized most DLBCL cases (70.59%, *p* = 0.001) [165].

Comparative analyses showed strong agreement between OCUS and CT in therapeutic monitoring (κ = 0.758), while OCUS required longer intervals to confirm complete remission than CT (6.01 ± 2.14 vs. 4.47 ± 1.84 months, *p* = 0.143) [165]. OCUS also demonstrated longer intervals than endoscopy (6.01 ± 2.14 vs. 4.71 ± 1.03 months, *p* = 0.088) [165]. These temporal discrepancies may stem from OCUS’s reduced capacity to distinguish residual lesions from post-treatment fibrotic changes [166, 167]. While OCUS demonstrates a certain potential for gastric lymphoma characterization and therapeutic monitoring, its clinical integration necessitates overcoming inherent limitations through standardized imaging protocols, advanced fibrosis-discrimination algorithms, and multicenter validation studies to optimize diagnostic concordance with gold-standard modalities. In addition, after intravenous contrast agent administration, the lymphoma is characterized by heterogeneous hyper-enhancement with delayed washout, reflecting tumor vascular heterogeneity [60], whereas related research is still under further exploration.

## Conclusion

OCUS and DCEUS have been gradually overcoming the challenges of ultrasound in the examinations of hollow viscera, which provide clinicians with an acceptable, multidimensional understanding of the characteristics of gastric tumors, and to a certain extent, promote the progress of gastric tumor monitoring, diagnosis and treatment decision-making. Compared to gastroscopy, EUS and enhanced CT, OCUS and DCEUS offer different advantages of non-invasiveness, non-ionizing radiation, low cost, low renal burden and real-time dynamic imaging. They can be used as effective supplementary diagnostic methods for GC in high-risk populations, and are expected to improve the detection rate of ESGC, especially in China.

This review systematically delineates the comprehensive synthesis of OCUS and DCEUS applications across the GC care continuum, encompassing screening, diagnostic confirmation, staging precision, and treatment response monitoring. Nevertheless, several limitations constrain their current utility. First, diagnostic accuracy still depends on the influence of operator experience and technical skills, which may reduce the reliability of the results. Second, although oral contrast agents can minimize intragastric gas interference, they cannot completely eliminate persistent gas-related artifacts. Third, in patients with GC who undergo NAT, a pathological complete response to the primary tumor may occur. However, fibrotic remodeling after necrosis and tumor-related inflammatory responses perpetuate gastric wall thickening, reducing gastric contrast ultrasonography’s staging accuracy. Fourth, gastric wall peristalsis is also a critical factor affecting the accuracy of GC staging by gastric contrast ultrasonography, especially ESGC.

As potentially effective and feasible technical approaches, future work should further conduct prospective large-sample studies on OCUS and DCEUS, and develop novel oral contrast agents and popularize their application. Concurrently, objective diagnostic indicators that are easy to standardize and promote should be established, and an intelligent assisted diagnosis system for gastric contrast ultrasonography can be developed to reduce the impact of examiners’ experience, improve diagnostic accuracy and efficiency, and provide high-quality imaging screening for physical examination institutions—thereby rapidly and comprehensively elevating screening and diagnostic capabilities. Real-time deep-learning–based probe or plane guidance can stabilize insonation and improve capture of arterial through late phases [168, 169]. Automated lesion and gastric-wall segmentation on gastric contrast ultrasonography cine loops with machine-extracted TIC metrics, referenced to adjacent normal wall, provides reproducible perfusion indices and reduces examiner subjectivity [170, 171]. During early adoption, synchronous tele-ultrasound supervision maintains diagnostic consistency and image quality, albeit with modestly longer scan time, thereby offering a practical bridge while local expertise is built [172]. With further exploration, OCUS and DCEUS are expected to become indispensable components in the GC diagnosis and treatment, enabling earlier and more accurate interventions to optimize patient outcomes and advance precision medicine for gastric tumors.

## Data Availability

The datasets are available from the corresponding authors.

## Abbreviations

AGC: Advanced GC
AI: Artificial intelligence
AT: Arrival time
BI: Basic intensity
CEUS: Contrast-enhanced ultrasonography
CT: Computed tomography
DCEUS: Double contrast-enhanced ultrasonography
DLBCL: Diffuse large B-cell lymphoma
EI: Enhanced intensity
ESGC: Early-stage GC
EUS: Endoscopic ultrasonography
GC: Gastric cancer
GISTs: Gastrointestinal stromal tumors
LNM: Lymph node metastasis
MALT: Mucosa-associated lymphoid tissue
MRI: Magnetic resonance imaging
NAT: Neoadjuvant therapy
NPV: Negative predictive value
OCUS: Oral contrast ultrasonography
PD-L1: Programmed death ligand-1
PI: Peak intensity
PPV: Positive predictive value
Su-RADS: Stomach Ultrasound Reporting and Data System
TIC: Time-intensity curve
VEGF: Vascular endothelial growth factor
